# Supplementary material for: Enhancing the Shelf Life of Sous-Vide Red Deer Meat with Piper nigrum Essential Oil: A Study on Antimicrobial Efficacy against Listeria monocytogenes
Source: Molecules. 2024 Sep 3;29(17):4179. doi: 10.3390/molecules29174179 (PMC11396834; doi:10.3390/molecules29174179)
Supplement: Supplementary file 1 [file molecules-29-04179-s001.zip › molecules-3179187-supplementary.pdf]

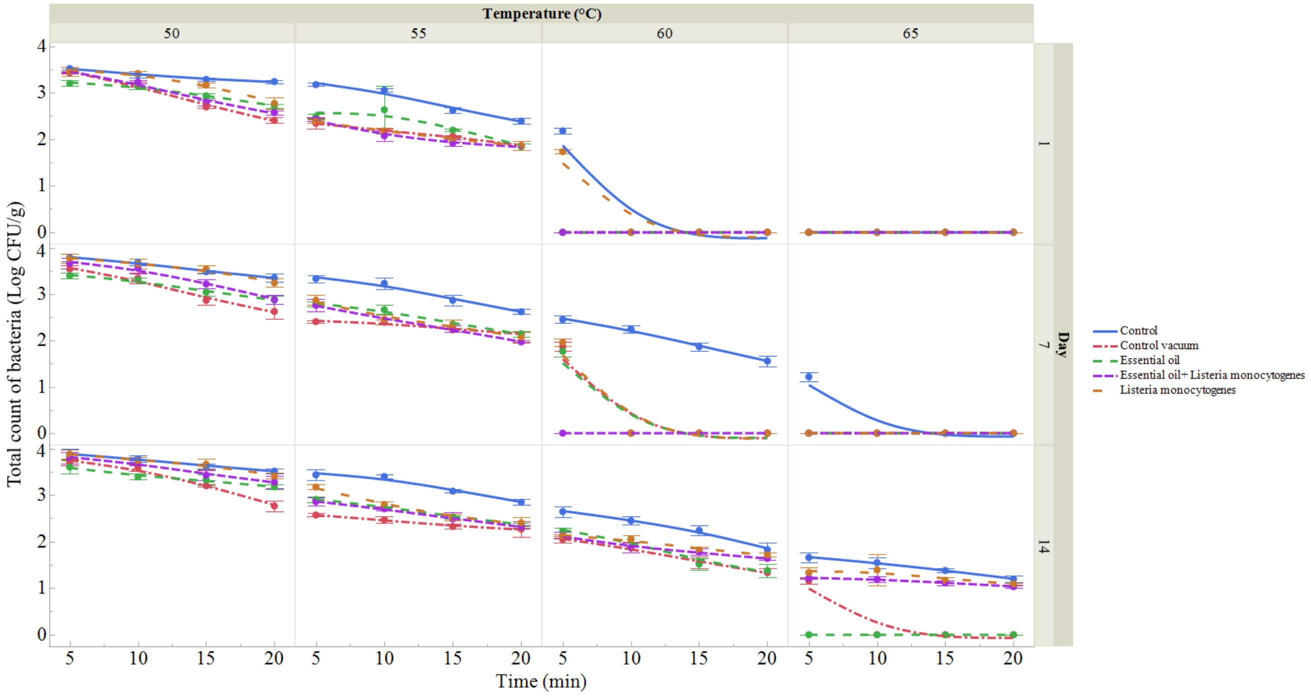

Figure S1. Total viable count (log CFU/g) of sous-vide red deer meat samples after storage 1, 7 and 14 days treated in a water bath at temperatures between 50 and 65 °C for 5 to 20 min. Data are the mean (bars indicate ± SD) of 3 red deer meat samples. Control: red deer meat samples placed in polyethylene bags without vacuum. Control vacuum: red deer meat samples vacuum-packed in polyethylene bags. Essential oil: red deer meat samples treated with 1% PNEO and vacuum-packed. *Listeria monocytogenes*: red deer meat samples inoculated with *L. monocytogenes* and vacuum-packed. Essential oil + *Listeria monocytogenes:* red deer meat samples treated with 1% PNEO and inoculated with *L. monocytogenes* and vacuum-packed.

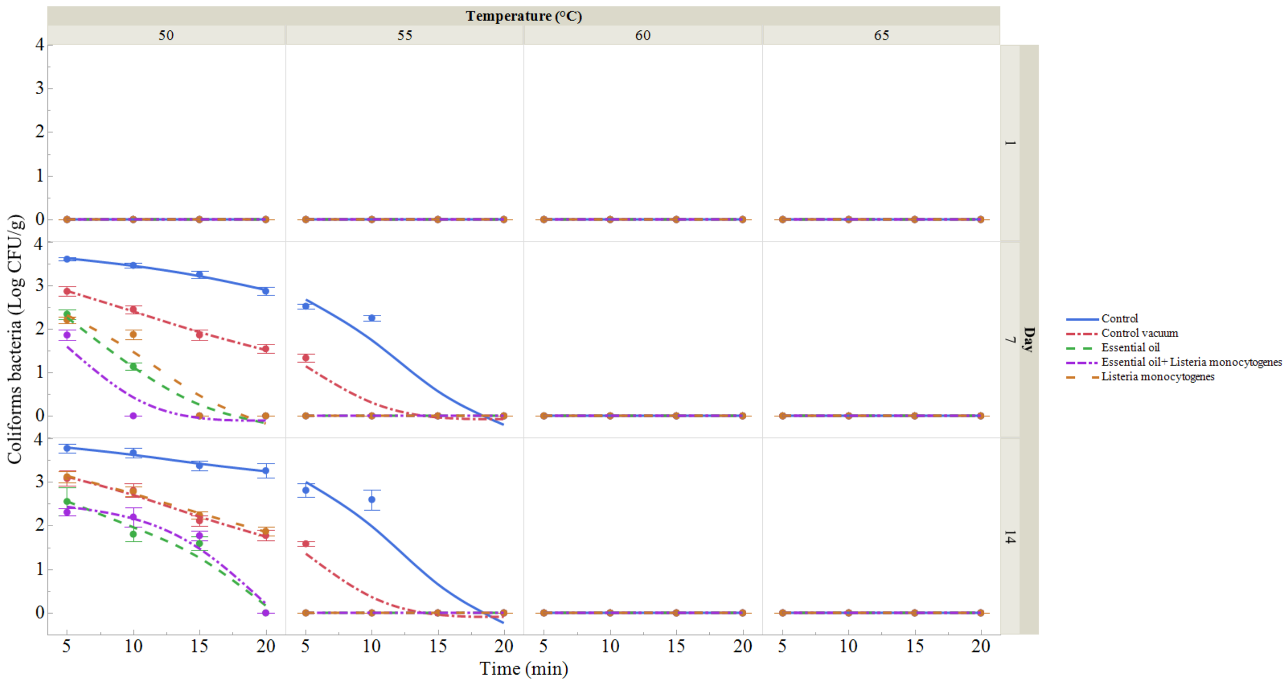

Figure S2. Total coliforms bacteria (log CFU/g) of sous-vide red deer meat samples after storage 1, 7 and 14 days treated in a water bath at temperatures between 50 and 65 °C for 5 to 20 min. Data are the mean (bars indicate ± SD) of 3 red deer meat samples. Control: red deer meat samples placed in polyethylene bags without vacuum. Control vacuum: red deer meat samples vacuum-packed in polyethylene bags. Essential oil: red deer meat samples treated with 1% PNEO and vacuum-packed. *Listeria monocytogenes*: red deer meat samples inoculated with *L. monocytogenes* and vacuum-packed. Essential oil + *Listeria monocytogenes:* red deer meat samples treated with 1% PNEO and inoculated with *L. monocytogenes* and vacuum-packed.

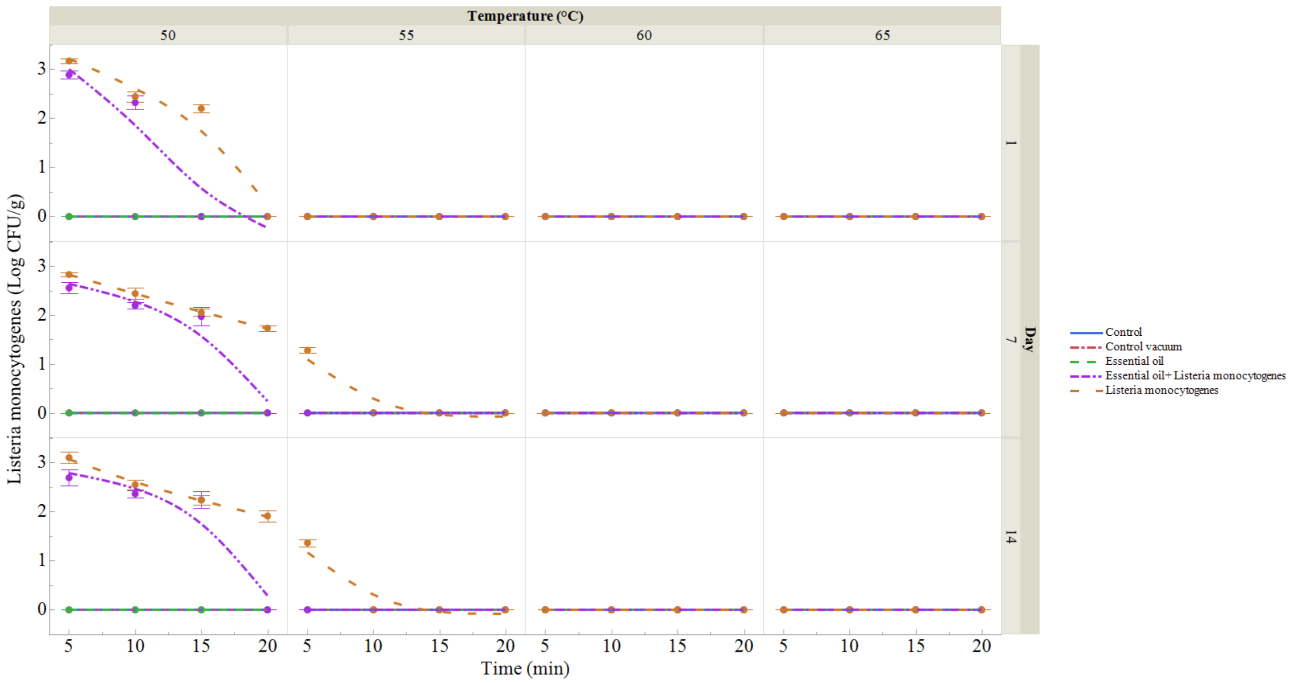

Figure S3. *L. monocytogenes* count (log CFU/g) of sous-vide red deer meat samples after storage 1, 7 and 14 days treated in a water bath at temperatures between 50 and 65 °C for 5 to 20 min. Data are the mean (bars indicate ± SD) of 3 red deer meat samples. Control: red deer meat samples placed in polyethylene bags without vacuum. Control vacuum: red deer meat samples vacuum-packed in polyethylene bags. Essential oil: red deer meat samples treated with 1% PNEO and vacuum-packed. *Listeria monocytogenes*: red deer meat samples inoculated with *L. monocytogenes* and vacuum-packed. Essential oil + *Listeria monocytogenes:* red deer meat samples treated with 1% PNEO and inoculated with *L. monocytogenes* and vacuum-packed.
